# Supplementary material for: The immediate treatment outcomes and cost estimate for managing clinical measles in children admitted at Mulago Hospital: A retrospective cohort study
Source: PLOS Glob Public Health. 2023 Jul 21;3(7):e0001523. doi: 10.1371/journal.pgph.0001523 (PMC10361502; doi:10.1371/journal.pgph.0001523)
Supplement: S1 File — (DOCX) [file pgph.0001523.s001.docx]

## S1 File. DATA ABSTRACTION FORM

Study ID number (3 digits)

**STUDY TITLE: IMMEDIATE TREATMENT OUT COMES AND THE COST ESTIMATE FOR MANAGING CLINICAL MEASLES IN CHILDREN 0-12 YEARS ADMITTED AT MULAGO HOSPITAL.**

**Principal investigator: Dr Namugga Barbara**

**Section A: Screening questions**

**Instructions: All responses should be YES for a chart to be included for review. If NO to any response, exclude the chart.**

**Tick the appropriate response**

|  | | **1** | Does the chart have an admission diagnosis of measles in 2018? | | | | | | | | | | **yes** | **no** | |
| --- | --- | --- | --- | --- | --- | --- | --- | --- | --- | --- | --- | --- | --- | --- | --- |
|  | |  |  | |  | |  | |  |  |  |  |  |  | |
|  | | **2** | Does the chart belong to a child between 0-12 years? | | | | | | | |  |  | **yes** | **no** | |
|  | |  |  | |  | |  | |  |  | |  |  |  | |
|  | | **3** | Does the chart have a documented Inpatient number, age and sex? (all the 3 | | | | | | | | | | **yes** | **no** | |
|  | |  | must be documented) | | |  |  |  |  |  |  |  |  |  | |
|  | |  |  | |  | |  | |  |  | |  |  |  | |
|  | | **4** | Did the patient have history of fever and rash plus any of: cough, coryza or | | | | | | | | | | **yes** | **no** | |
|  | |  | conjunctivitis documented? | | |  |  |  |  |  |  |  |  |  | |
|  | |  |  | |  |  |  |  |  |  |  |  |  |  | |
|  | |  |  | |  |  |  |  |  |  |  |  |  |  | |
| **Section B: Outcome Questions** | | | | | |  |  |  |  |  |  |  |  |  | |
|  | |  | | |  |  |  |  |  |  |  |  |  |  | |
|  | | Participant Hospital number **(6** | | |  |  |  |  |  |  |  |  |  |  | |
|  | | **digits)** | | |  | …………………… | | | | |  |  |  |  | |
|  | |  | | |  |  |  | |  |  |  |  |  |  | |
|  | | Research Assistant’s code | | |  | …………………… | | | | |  |  |  |  | |
|  | |  | | |  |  |  |  |  |  |  |  |  |  | |
|  | | B1.Date of admission | | |  |  |  |  |  |  |  |  |  |  | |
|  | | **(dd/mm/yy)** | | |  | …………./……………/………… | | | | | | |  |  | |
|  | |  | | |  |  |  |  |  |  |  |  |  |  | |
|  | | B2.Date of discharge/Death | | |  | / | | / | |  | . |  |  |  | |
|  | |  |  | |  |  |  |  |  |  |  |  |  |  | |
|  | |  |  | |  |  |  |  |  |  |  |  |  |  | |
| **(dd/mm/yy)** | | |  | | | | | | | | | | |  |  |
|  | | |  | | | | | | | | | | |  |  |
| B3.Duration of stay in hospital | | |  | | | | | | | | | | |  |  |
| **(count from date of** | | |  | | | | | | | | | | |  |  |
| **admission to date of** | | |  | | | | | | | | | | |  |  |
| **discharge)** | | | ……………………….(days) | | | | | | | | | | |  |  |
|  | | |  |  |  |  |  |  |  |  |  |  |  |  |  |
|  | | |  | | | | | | | | | | |  |  |
| B4.Tick vital status at | | | Alive………..1 | | | | | | | | | | |  |  |
|  | | |  |  |  |  |  |  |  |  |  |  |  |  |  |
| discharge | | | Dead………...2 | | | | | | | | | | |  |  |
|  | | |  |  |  |  |  |  |  |  |  |  |  |  |  |
|  | | |  | | | | | | | | | | |  |  |

| NO. | QUESTIONS AND FILTERS | RESPONSES |
| --- | --- | --- |
|  |  |  |
| B5 | What was the child’s age at | ……………….years old |
|  | admission? | ……………..months if less than 1 year |
|  |  |  |
|  |  |  |
| B6 | What is the sex of the child? | 1=Male |
|  | (**tick what applies)** | 2=female |
|  |  |  |
| B7 | Tick all the symptoms/signs that | Cough………………………………1 |
|  | the child presented with in hospital | Flue (coryza)………………………..2 |
|  |  |  |
|  | during admission? | Conjunctivitis (red eyes)…………....3 |
|  |  |  |
|  |  | Rash………………………………...4 |
|  |  | Fever………………………………..5 |
|  |  | Kopliks spots………………………..6 |
|  |  | Diarrhoea……………………………7 |
|  |  | Vomiting……………………………8 |
|  |  | Convulsions………………………....9 |
|  |  | Difficulty in breathing……10 |
|  |  | Loss of consciousness…….11 |
|  |  |  |

|  |  | Other (specify…………….12 |
| --- | --- | --- |
|  |  | 1………………………. |
|  |  | 2……………………… |
|  |  |  |
| B8 | Was the child immunised against | 1= Yes |
|  | measles? **( tick what applies)** | 2= No |
|  |  |  |
|  |  | 3=Unknown/not recorded. |
|  |  | 4=less than 9 months (not due) |
|  |  |  |
| B9 | What long standing illness was | HIV…………………1 |
|  |  |  |
|  | recorded during admission period? | Cerebral palsy……….2 |
|  |  |  |
|  | **(Tick all that apply)** | Tuberculosis…………3 |
|  |  |  |
|  |  | Heart disease………...4 |
|  |  | Kidney disease………5 |
|  |  | Liver disease…………7 |
|  |  | Sickle cell disease……8 |
|  |  | If others specify………9 |
|  |  | 1…………………… |
|  |  | 2…………………….. |
|  |  |  |
| B10 | **Tick all** the diagnoses | **Measles with;** |
|  |  |  |
|  | (complications) **during the** | Severe |
|  | **admission period** | Pneumonia……………………………..........1 |
|  |  | Gastroenteritis( acute watery diarrhoea)…….2 |
|  |  | Otitis media………………………………….3 |
|  |  | Conjunctivitis………………………………..4 |
|  |  | Malnutrition………………………………….5 |
|  |  | Encephalitis………………………………….6 |
|  |  |  |


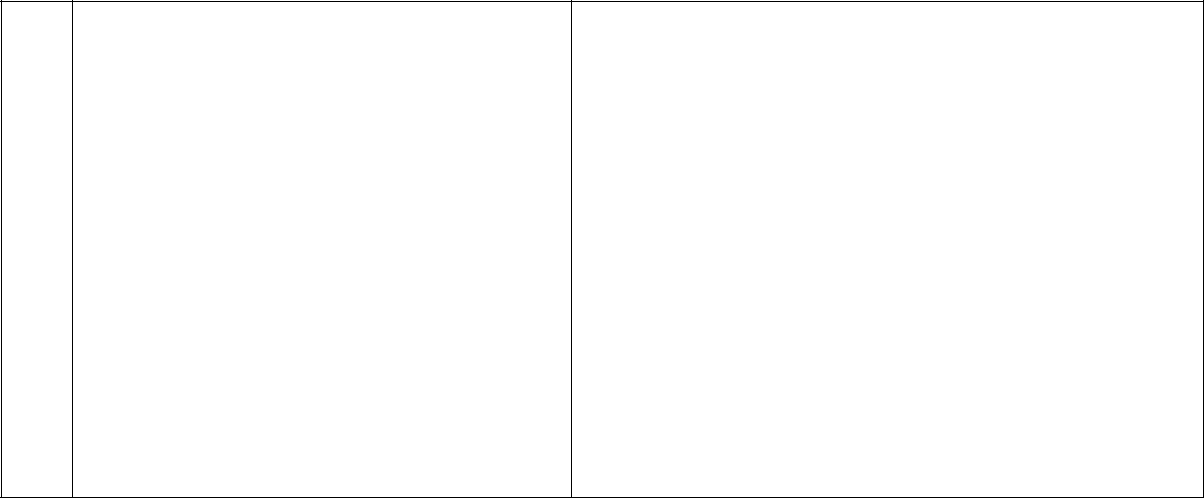


Shock ………………………………………..7

Anaemia……………………………………...8

Others (list

them)…………………………………………9

1………………………….

2………………………….

3……………………….....

**SECTION C: COST ANALYSIS**

| **No** | |  | **Items** | | **Information to be obtained** | | **Information to be obtained** | |
| --- | --- | --- | --- | --- | --- | --- | --- | --- |
|  |  |  |  |  | **from the patient’s file** | | **from the price list** | |
|  |  |  |  |  |  |  |  |  |
|  | **C** |  |  | **Blood Tests** | **Tick if** | **Number of** | **Unit cost** | **Total cost** |
|  |  |  |  |  | **requested by** | **times done** | **(Ug.Shs)** |  |
|  |  |  |  |  | **the clinician** | **during** |  |  |
|  |  |  |  |  |  | **admission** |  |  |
|  |  |  |  |  |  |  |  |  |
|  | C1 |  |  | Blood smear(B/S) |  |  |  |  |
|  |  |  |  |  |  |  |  |  |
|  | C2 |  |  | Complete Blood |  |  |  |  |
|  |  |  |  | Count (CBC) |  |  |  |  |
|  |  |  |  |  |  |  |  |  |
|  | C3 |  |  | Liver Function |  |  |  |  |
|  |  |  |  | Tests(LFTs) |  |  |  |  |
|  |  |  |  |  |  |  |  |  |
|  | C4 |  |  | Renal Functional |  |  |  |  |
|  |  |  |  | Tests (RFTs) |  |  |  |  |
|  |  |  |  |  |  |  |  |  |
|  | C5 |  |  | Other tests, specify |  |  |  |  |
|  |  |  | 1 | |  |  |  |  |
|  |  |  | 2 | |  |  |  |  |
|  |  |  | 3 | |  |  |  |  |
|  |  |  | 4 | |  |  |  |  |
|  |  |  |  |  |  |  |  |  |
|  |  |  |  | Sub total |  |  |  |  |
|  |  |  |  |  |  |  |  |  |

| **No** | |  | **Items** | **Information to be obtained from the** | |  | **Information to be** | |
| --- | --- | --- | --- | --- | --- | --- | --- | --- |
|  |  |  |  | **patient’s file** |  |  | **obtained from the price list** | |
|  |  |  |  |  |  |  |  |  |
|  |  |  |  |  |  |  |  |  |
|  |  |  | **Radiology** | **Tick if requested by** | **Number of** |  | **Unit cost** | **Total cost** |
|  |  |  |  | **the clinician** | **times done** |  | **(Ug.Shs)** |  |
|  |  |  |  |  | **during** |  |  |  |
|  |  |  |  |  | **admission** |  |  |  |
|  |  |  |  |  |  |  |  |  |
|  | C7 |  | Chest X-Rays |  |  |  |  |  |
|  |  |  |  |  |  |  |  |  |
|  | C8 |  | Abdominal |  |  |  |  |  |
|  |  |  | ultrasound scan |  |  |  |  |  |
|  |  |  |  |  |  |  |  |  |
|  | C9 |  | Brain CT scan |  |  |  |  |  |
|  |  |  |  |  |  |  |  |  |
|  |  |  | Sub total |  |  |  |  |  |
|  |  |  |  |  |  |  |  |  |

| **ITEM** | **Information to be obtained from the patient file** | | | | **Information to be obtained** | |
| --- | --- | --- | --- | --- | --- | --- |
|  |  |  |  |  | **from the price list** | |
|  |  |  |  |  |  |  |
|  | **Indicate** |  | **Number of** | **Total number of** | **Unit cost** | **Total cost** |
| **Treatment** | **whether** | **Dosage** | **days** | **tablets/capsules/** | **Ug.Shs** |  |
|  | **caps/injection** |  | **prescribed/** | **ampules/vials** |  |  |
|  |  | **and** |  |  |  |  |
|  | **s/tabs/** |  | **given** |  |  |  |
|  |  | **freque** |  |  |  |  |
|  |  |  |  |  |  |  |
|  |  | **ncy** |  |  |  |  |
|  |  |  |  |  |  |  |
| C10.Antibi |  |  |  |  |  |  |
| Otics |  |  |  |  |  |  |
| 1 |  |  |  |  |  |  |
| 2 |  |  |  |  |  |  |
| 3 |  |  |  |  |  |  |
| 4 |  |  |  |  |  |  |
|  |  |  |  |  |  |  |
| C11.Analg |  |  |  |  |  |  |
| esics/ |  |  |  |  |  |  |
| antipyretics |  |  |  |  |  |  |
| 1 |  |  |  |  |  |  |
| 2 |  |  |  |  |  |  |
| 3 |  |  |  |  |  |  |
|  |  |  |  |  |  |  |
| C12.Others |  |  |  |  |  |  |
| specify |  |  |  |  |  |  |
| 1 |  |  |  |  |  |  |
| 2 |  |  |  |  |  |  |
| 3 |  |  |  |  |  |  |
|  |  |  |  |  |  |  |


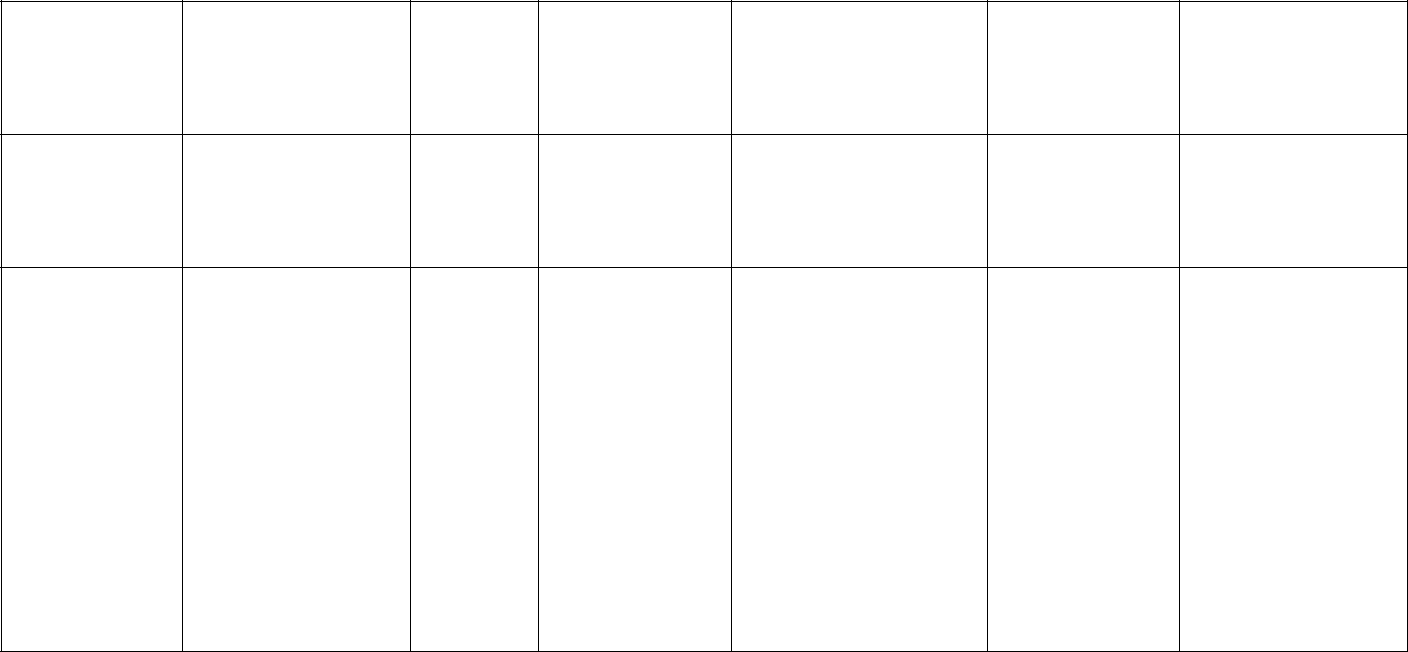


C13.Zinc tablets

sulphate

C14.Vitami capsules

n A

C15.Fluids **(*Specify if***

***ORS or IV****)*

1

2

3

|  | **ITEM;** | | | **Information to be obtained from the** | | | | **Information to be obtained** | |
| --- | --- | --- | --- | --- | --- | --- | --- | --- | --- |
|  |  |  |  | **patient’s chart** |  |  |  | **from the price list** | |
|  |  |  |  |  |  |  |  |  |  |
|  | **Supplies** | | |  |  |  |  |  |  |
|  |  |  |  |  |  |  |  |  |  |
|  | Gloves | | | What was the | How many |  | Total number | Unit cost | Total cost |
|  |  |  |  | frequency of the | days was the |  | of gloves |  |  |
|  |  |  |  | prescribed IV | medicine to |  | (pairs) |  |  |
|  |  |  |  | treatment per | be given |  | **(frequency** |  |  |
|  |  |  |  | day |  |  | **of IV** |  |  |
|  |  |  |  |  |  |  | **treatment*n** |  |  |
|  |  |  |  |  |  |  | **umber of** |  |  |
|  |  |  |  |  |  |  | **days** |  |  |
|  |  |  |  |  |  |  | **treatment** |  |  |
|  |  |  |  |  |  |  | **was given** |  |  |
|  |  | |  |  |  |  |  |  |  |
|  | C16**.Number of pairs** | | |  |  |  |  |  |  |
|  |  |  |  |  |  |  |  |  |  |
|  | **of gloves** | | |  |  |  |  |  |  |
|  |  | |  |  |  |  |  |  |  |
|  | Cannulas | | | **( take an average of 2** | |  |  |  |  |
|  |  |  |  | **cannulas per patient)** | |  |  |  |  |
|  |  | |  |  |  |  |  |  |  |
|  | C17.**Number of** | | |  |  |  |  |  |  |
|  |  |  |  |  |  |  |  |  |  |
|  | **cannulas** | | |  |  |  |  |  |  |
|  |  | |  |  |  |  |  |  |  |
|  | ***C18. Syringes 5ML*** | | |  |  |  |  |  |  |
|  |  |  |  |  |  | |  |  |  |

|  | How many IV drugs | | Indicate the total | How many |  | Total number | Unit cost of | Total cost of syringes |
| --- | --- | --- | --- | --- | --- | --- | --- | --- |
|  | were prescribed? | | number of times | days were |  | of | 5ml syringe |  |
|  |  |  | for the IV | prescribed |  | syringes=no. |  |  |
|  |  |  | treatment per | for the IV |  | IV treatment* |  |  |
|  |  |  | day | treatment |  | frequency*nu |  |  |
|  |  |  |  |  |  | mber of days |  |  |
|  |  |  |  |  |  |  |  |  |
|  |  |  |  |  |  |  |  |  |
|  |  |  |  |  |  |  |  |  |
|  | Oxygen | | **( tick if** | How many |  | Unit cost per day ***(*** | | Total cost |
|  |  |  | **prescribed and** | days was |  | ***information from price list)*** | |  |
|  |  |  | **indicate** | oxygen |  |  |  |  |
|  |  |  | **number of days** | prescribed? |  |  |  |  |
|  |  |  | **if recorded )** |  |  |  |  |  |
|  |  | |  |  |  |  |  |  |
|  | C19.Number of days | |  |  |  |  |  |  |
|  |  |  |  |  |  |  |  |  |
|  | oxygen was | |  |  |  |  |  |  |
|  | prescribed | |  |  |  |  |  |  |
|  |  |  |  |  |  |  |  |  |

|  | **Giving set** | | Tick if | Total number of | | giving sets | | Unit cost | Total cost |
| --- | --- | --- | --- | --- | --- | --- | --- | --- | --- |
|  |  |  | prescribed | (**assume 1 giving set for all** | | | |  |  |
|  |  |  | IV fluids | **who received 1V fluids)** | | | |  |  |
|  |  | |  |  |  |  |  |  |  |
|  | C20.Response | |  |  |  |  |  |  |  |
|  |  |  |  |  |  | |  |  |  |
|  | Nasogastric tube | | Tick if | Assume 1 nasogastric tube per | | | | Unit cost | Total cost |
|  |  |  | prescribed | admission if prescribed. | | | |  |  |
|  |  | |  |  |  |  |  |  |  |
|  | C21.Response | |  |  |  |  |  |  |  |
|  |  |  |  |  |  |  |  |  |  |
|  | **Nutritional** | | **tick if** |  | **indicate** |  | Duration | Unit cost | Total cost |
|  | **supplements** | | **prescribed** |  | **frequency** |  | (days) |  |  |
|  |  |  |  |  |  | |  |  |  |

|  | C22.F75 | |  |  |  |  |  |
| --- | --- | --- | --- | --- | --- | --- | --- |
|  |  |  |  |  |  |  |  |
|  | C23.F100 | |  |  |  |  |  |
|  |  |  |  |  |  |  |  |
|  | C24.RTUF(ready to | |  |  |  |  |  |
|  |  |  |  |  |  |  |  |
|  | use feeds) | |  |  |  |  |  |
|  |  | |  |  |  |  |  |
|  | **C25.**Indicate the flat | |  | | | | **2,000** |
|  |  |  |  |  |  |  |  |
|  | fee to cover for | |  | | | |  |
|  | strapping, swabs | |  | | | |  |
|  | disinfectant ( for the | |  | | | |  |
|  | period of admission**)** | |  | | | |  |
|  |  | |  |  |  |  |  |
|  | **Subtotal cost** | |  | | | |  |
|  |  | |  | | |  |  |
|  | **Grand total** | |  | | |  |  |
|  |  |  |  |  |  |  |  |

**SECTION D: DIRECT NON HEALTH CARE COST TOOL**

**STUDY TITLE: *IMMEDIATE TREATMENT OUT COMES AND THE COST* *ESTIMATE FOR MANAGING CLINICAL MEASLES IN CHILDREN 0-12 YEARS ADMITTED AT MULAGO HOSPITAL*.**

Principal Investigator: Dr Namugga Barbara

Interviewee:……………………………………………………………….

Date of interview.dd/mm/yy………./………../………

Costs to be obtained from the accounts department in Mulago Hospital

1. **Please complete the following regarding to human resource professionals at acute care unit.**

|  |  |  | Title | No. | Monthly | Daily |  | Consultation | Nursing | Total cost per |
| --- | --- | --- | --- | --- | --- | --- | --- | --- | --- | --- |
|  |  |  | /cadres |  | salary | salary |  | time for doctor | time | day (Ug.Shs) |
|  |  |  |  |  | (Ug.Shs) |  |  | ***Assuming a Dr*** | ***Assuming*** |  |
|  |  |  |  |  |  |  |  | ***spends 30min*** | ***a nurse*** |  |
|  |  |  |  |  |  |  |  | ***per patient*** | ***spends I*** |  |
|  |  |  |  |  |  |  |  | ***(0.5hours)*** | ***hour per*** |  |
|  |  |  |  |  |  |  |  |  | ***patient per*** |  |
|  |  |  |  |  |  |  |  |  | ***day*** |  |
|  |  | |  |  |  |  |  |  |  |  |
|  | D1 | |  |  |  |  |  |  |  |  |
|  |  |  |  |  |  |  |  |  |  |  |
|  | D2 | |  |  |  |  |  |  |  |  |
|  |  |  |  |  |  |  |  |  |  |  |
|  | D3 | |  |  |  |  |  |  |  |  |
|  |  |  |  |  |  |  |  |  |  |  |
|  | D4 | |  |  |  |  |  |  |  |  |
|  |  |  |  |  |  |  |  |  |  |  |
|  | D5 | |  |  |  |  |  |  |  |  |
|  |  |  |  |  |  |  |  |  |  |  |
|  | D6 | |  |  |  |  |  |  |  |  |
|  |  |  |  |  |  |  |  |  |  |  |

***B; Please complete the following regarding to human resource professionals at ward 1C***

|  | Title | | | No. | Monthly | Daily salary | Consultatio | Nursing | Total cost per day |
| --- | --- | --- | --- | --- | --- | --- | --- | --- | --- |
|  | /cadres | | |  | salary | (Ug.Shs) | n time for | time | (Ug.Shs) |
|  |  |  |  |  | (Ug.Shs) |  | doctor | ***Assuming a*** |  |
|  |  |  |  |  |  |  | ***Assuming a*** | ***nurse*** |  |
|  |  |  |  |  |  |  | ***Dr spends*** | ***spends*** |  |
|  |  |  |  |  |  |  | ***15min per*** | ***0.5hours*** |  |
|  |  |  |  |  |  |  | ***patient*** | ***per patient*** |  |
|  |  |  |  |  |  |  | ***(0.25hours)*** | ***per day*** |  |
|  |  | |  |  |  |  |  |  |  |
|  | D8 | | |  |  |  |  |  |  |
|  |  |  |  |  |  |  |  |  |  |
|  | D9 | | |  |  |  |  |  |  |
|  |  |  | |  |  |  |  |  |  |
|  | D10 | | |  |  |  |  |  |  |
|  |  | |  |  |  |  |  |  |  |
|  | D11 | | |  |  |  |  |  |  |
|  |  | |  |  |  |  |  |  |  |
|  | D12 | | |  |  |  |  |  |  |
|  |  | |  |  |  |  |  |  |  |
|  | D13 | | |  |  |  |  |  |  |
|  |  |  |  |  |  |  |  |  |  |

1. **What are the other overhead costs associated with treating each case of measles in Mulago Hospital?**

|  | No. |  | ITEM | Unit cost per patient | Total cost (Ug.Shs) |
| --- | --- | --- | --- | --- | --- |
|  |  |  |  | (Ug.Shs) |  |
|  |  |  |  |  |  |
|  | D14 |  | Water |  |  |
|  |  |  |  |  |  |
|  | D15 |  | Electricity |  |  |
|  |  |  |  |  |  |
|  | D16 |  | Security |  |  |
|  |  |  |  |  |  |
|  | D18 |  | Equipment maintenance |  |  |
|  |  |  |  |  |  |
|  | D19 |  | Meals |  |  |
|  |  |  |  |  |  |
|  | D20 |  | Stationary |  |  |
|  |  |  |  |  |  |
|  | D21 |  | Cleaning |  |  |
|  |  |  |  |  |  |
|  | D22 |  | Accommodation |  |  |
|  |  |  |  |  |  |
|  | D23 |  | List others |  |  |
|  |  |  |  |  |  |
|  |  |  | 1 |  |  |
|  |  |  |  |  |  |
|  |  |  | 2 |  |  |
|  |  |  |  |  |  |
|  |  |  | 3 |  |  |
|  |  |  |  |  |  |
|  |  |  | Total cost |  |  |
|  |  |  |  |  |  |
