# Supplementary material for: The immediate treatment outcomes and cost estimate for managing clinical measles in children admitted at Mulago Hospital: A retrospective cohort study
Source: PLOS Glob Public Health. 2023 Jul 21;3(7):e0001523. doi: 10.1371/journal.pgph.0001523 (PMC10361502; doi:10.1371/journal.pgph.0001523)
Supplement: S1 Table — (DOCX) [file pgph.0001523.s002.docx]

S2 Table. Immunization status of 185 children who had multiple complication

| **Immunization status** | **Frequency** | **Percentage** |
| --- | --- | --- |
| Yes | 50 | 27.0% |
| Unknown | 48 | 25.9% |
| Not due | 33 | 17.8% |
| No | 54 | 29.2% |
| **Grand Total** | **185** | **100.00%** |
