# Supplementary material for: The immediate treatment outcomes and cost estimate for managing clinical measles in children admitted at Mulago Hospital: A retrospective cohort study
Source: PLOS Glob Public Health. 2023 Jul 21;3(7):e0001523. doi: 10.1371/journal.pgph.0001523 (PMC10361502; doi:10.1371/journal.pgph.0001523)
Supplement: S2 Table — (DOCX) [file pgph.0001523.s003.docx]

**S3 Table. Clinical and demographic characteristics of the 30 patients with clinical measles who died during hospital stay.**

| **Variable** | **Category** | **Frequency (n=30)** | **Percentage %** |
| --- | --- | --- | --- |
| **Sex** | Male | 17 | 57.0 |
|  | Female | 13 | 43.0 |
| **Age ^a^** | < 1 year | 21 | 70.0 |
|  | ≥1 year | 9 | 30.0 |
| **Immunization status** | Yes | 4 | 13.0 |
|  | No | 13 | 44.0 |
|  | Not documented | 7 | 23.0 |
|  | Not due | 6 | 20.0 |
| **Immunisation status ≤ 1 year** | Yes | 4 | 13.3 |
|  | No | 7 | 23.3 |
|  | Not documented | 4 | 13.3 |
|  | Not due | 6 | 20.1 |
| **Hospital days** | < 7 | 26 | 86.0 |
|  | ≥7 | 4 | 14.0 |
| **Co-morbidities at admission** | Yes | 5 | 17.0 |
|  | No | 25 | 83.0 |

**^a^** *Only 1 child aged over 5 years died*
