# Supplementary material for: The immediate treatment outcomes and cost estimate for managing clinical measles in children admitted at Mulago Hospital: A retrospective cohort study
Source: PLOS Glob Public Health. 2023 Jul 21;3(7):e0001523. doi: 10.1371/journal.pgph.0001523 (PMC10361502; doi:10.1371/journal.pgph.0001523)
Supplement: S3 Table — (DOCX) [file pgph.0001523.s004.docx]

S4 Table . Demographic characteristics of 60 patients who stayed longer than 7 days

| **Variable** | **Category** | **Frequency (n=60)** | **Percentage** |
| --- | --- | --- | --- |
| **Sex** | Male | 36 | 60.0 |
|  | Female | 24 | 40.0 |
| **Age** | < 1years | 46 | 77.0 |
|  | ≥1years | 14 | 23.0 |
| **Immunization status** | Yes | 14 | 23.0 |
|  | No | 18 | 30.0 |
|  | Not documented | 15 | 25.0 |
|  | Not due | 13 | 22.0 |
| **Comorbidities** | Yes | 6 | 10.0 |
|  | No | 54 | 90.0 |
