# Supplementary material for: The immediate treatment outcomes and cost estimate for managing clinical measles in children admitted at Mulago Hospital: A retrospective cohort study
Source: PLOS Glob Public Health. 2023 Jul 21;3(7):e0001523. doi: 10.1371/journal.pgph.0001523 (PMC10361502; doi:10.1371/journal.pgph.0001523)
Supplement: Attachment — Submitted filename: RESPONSE TO REVIEWERS.docx [file pgph.0001523.s005.docx]

18^th^ May 2023

**The Editor,**

**PLOS Global Public Health Journal**

RE: Resubmission of an original article titled **“The immediate treatment outcomes and cost estimate for managing clinical measles in children admitted at Mulago Hospital: a retrospective cohort study”**

We are delighted that PLOS Global Public Health considered our manuscript for review. We have given careful consideration to the comments from the reviewers and editor, and have addressed them all.

Attached is a point-by-point explanation of how we have addressed the concerns and revised our manuscript.

In addition, we had forgotten to include the statistician (Leonard Manirakiza) as one of the authors and he has been added in the amended submission.

We look forward to a favorable opinion from the editor.

Yours sincerely,

Dr Namugga Barbara,

on behalf of all the authors

**Responses to reviewers’ comments**

Reviewer #1: This a relevant and timely topic as the world is experiencing many measles outbreaks in the COVID era. Evaluating the cost of treatment for preventable diseases provides a solid evidence-based argument to strengthen preventive strategies. However, for a more robust methodologic approach and better clarity, we offer those comments:
Objective
- For more clarity, please precise the study period in the objective. And specify that the "average cost" was determined.
Response ; The study period, i.e. 2018, has been included in the study objective in the last paragraph of the introduction section. We have also specified that “average cost” was determined, as suggested by the reviewer.

Method
- Why is the study defined as a single cohort study? The method and data presented are more in favor of a transversal study, not a longitudinal approach. If there is additional information that can support the Cohort aspect, please share.

RESPONSE; We considered this to be a cohort study because for each child considered in the analysis, data collected spanned the period of admission, to discharge/death. The word “single” has been deleted (methods section of the abstract), as we consider this unnecessary.

- The study procedures (Section Method) stated: “Market prices were used for those items that were not in the price list.” As it is a retrospective study, we wonder if there were any fluctuations between the current prices (prices during the data collection) and those in 2018. If yes, which one was used, and how did the authors avoid bias considering this outcome? The clarity of the study will benefit from that precision.

RESPONSE: We appreciate the reviewer for this observation. We agree that fluctuations in prices could have occurred. For those prices that had changed, the deflation rate was used to adjust the prices. Using the current Consumer Price Indices (CPI) and for 2018, the median unit prices were adjusted for inflation/deflation.

- It would help the readers’ comprehension if treatments cost was defined. What that variable included? (IV, drugs, equipment utilization…)? Additionally, it is better to keep the same classification of direct costs in both methods and results. In the Methods, the direct costs are defined as direct health care costs (drugs, supplies, and investigations)

Response: Treatment costs included cost of drugs and supplies. This has been defined in the second paragraph of the study procedures section. We confirm that the classification of direct costs in both methods and results has been maintained.

Results
- In the results section, “Direct health care costs were divided into three categories, i.e., blood tests, radiology, and treatment costs.” Blood tests and radiology can be labeled as investigations; drugs can be considered treatment. Are supplies included in the treatment group? The reader should not have to guess.

Response: This has been clarified in the third paragraph of the study procedure section. Treatment costs included drugs and supplies. Investigations included blood tests and radiological tests.

Discussion
- When considering the study objectives and the results presented, we realize that this study explores not only the direct costs as stated in the discussion. I would suggest removing the word "direct."
Response : The word ‘direct’ has been deleted in the first paragraph of the discussion section.

General comment
- It is unclear if the authors used only the clinical admission diagnosis of measles to select the study sample. If yes, it could have been interesting to know the proportion of confirmed cases and compare the costs between confirmed cases and suspect cases.

Response: We only used the clinical diagnosis of measles for the study. In Uganda, measles cases are not routinely confirmed by laboratory tests as the test is quite expensive and not easily accessible. A clinical diagnosis is many times used, therefore the authors used only the clinical diagnosis of measles. It is therefore not possible to compare the costs between the confirmed cases and suspects. We acknowledge that this would have been an interesting comparison to make.

Reviewer 2

Reviewer #2: Overall:
The paper is setting out to identify the direct and indirect clinical costs of managing measles cases, which it does ($114.5 USD average cost estimate in reference to $0.86 cost of measles vaccination). From here the authors go forward to say that these high costs of care are “strong ground to base on to promote vaccination,” however the data presented in this paper shows that immunization status has no statistical difference on direct hospital costs incurred ($36.49 immunized patient, $35.40 non-immunized patient). A more accurate conclusion should state that complications/comorbidities are present in a majority of fatal measles cases and comorbid cases carry a higher cost to the healthcare system.

Response: We appreciate the reviewer for this suggestion. We have edited our conclusion accordingly, and it now reads, “Complications are common in majority of fatal measles cases and these carry a high cost to the healthcare system.”

Comments on Introduction:
1. Edit hard immunity to herd immunity.

Response: This has been edited to herd immunity under the introduction.

Comments on Study Procedure:
2. Participant charts were identified from hospital diagnosed measles cases but some charts did not meet criterion for clinical measles and therefore were excluded. Although WHO Guidelines are referenced, it would be good for the reader to have an idea of the study’s inclusion/exclusion criteria in more detail.

Response: The inclusion/exclusion criteria is stated in the second paragraph of the study procedure section. Inclusion criteria was all available retrieved charts of children 0-12 years admitted with clinical measles from 1^st^January 2018 to 31st December 2018. Exclusion criteria included, a chart with a documented diagnosis of measles and yet does not have the clinical definition of measles as fever, maculopapular rash with either cough, coryza, or conjunctivitis. In addition, all those charts without documented inpatient number, age, sex, were excluded, as well as charts with illegible handwriting.

3. Age of inclusion is mentioned in the abstract, it is never mentioned in the Study Procedure section.

Response : The age of inclusion ( 0-12 years) has been added in the second paragraph of the study procedure section.

This is a study to establish the costs of managing measles cases, but there are no methods listed describing how non health care costs were actually determined. For security and staff, were the total number of staff salaries per day determined then divided by the daily number of patients in hospital?

Response: Further detail on how direct non health care costs were determined has been provided in the study procedure section.

Costs were classified as direct health care (treatment costs; (drugs and supplies) and investigations (blood tests and radiology tests) and direct non health care /overhead costs (security, salaries, water, electricity). These direct non health care costs were obtained from the hospital planning unit / accounts department and costed per day per patient. Monthly salaries of staff who work on wards where measles patients are admitted were obtained from the hospital accounts department and daily salaries were calculated. This was then divided by the average number of patients seen per day to get the average cost. Unit costs were obtained using the price list from the hospital. Market prices were used for those items that were not in the price list. Total costs per patient were calculated and average costs determined. All costs incurred were assumed to have been received by the patient since they would be required in the management of measles.

Does measles case coverage necessitate a higher staff per patient ratio

Response: Measles case coverage does not necessitate a higher staff per patient ratio. Such costs are shared across with other patient disease cases.

For utilities, is this a hospital that relies on generator electricity with high fuel costs or is there provision of consistent electricity?

Response: The hospital mainly relies on electricity which is consistent but also has a standby generator on the rare occasion that electricity goes off.

5. Data Abstraction Form (DAFs) could be shared in supplemental materials.

Response: This has been shared as supplementary material (S1).

Results:
6. No information is given about the Immunization status Not Due. A quick Google search shows me that US CDC guidelines for measles immunizations are 12-15 months, WHO guidelines are 9 months. This is very relevant information seeing that age range for participants is 0-12 years, the median age of participants is 1.0 year, a majority of patients are aged less than 1 year. Please provide justification, in terms of health outcomes, why patients in the Not Due category do not fall in the non-immunized category.

Response: Whereas children younger than 9months can still get measles, the WHO and ministry of Health recommendation for measles vaccination in the country is at 9 months. Those who were not due for measles immunization were below 9 months of age. We are of the opinion that classifying those “not due” together with the “non-immunized” may be misleading. Those who are “non-immunized” would otherwise have been eligible for immunisation, but did not get the vaccine, and this is the proportion that public health measures should particularly target in order to reduce on the risk of measles infection.

7. Please provide information/justification why Immunization status Unknown was not considered as a case with missing documentation and therefore subject to exclusion from the study.

Response : “immunization status Unknown” was not considered in the exclusion because the study was looking at the cost of managing a measles case and treatment outcomes regardless of the immunization status. In addition, 25.8% of charts retrieved had unknown immunization status and their exclusion would impact on the sample size attainment.

8. The text states that Table 1 displays 30 deaths and 237 discharges, but Table 1 does not contain this information.

Response: This information has been provided in table 1. An extract of this is shared here.

| **Duration of hospital stay** | Median (IQR) | 4.0 (3.0-7.0) |
| --- | --- | --- |
|  | 0-7 | 207 (78 %) |
|  | 8-15 | 50 (19 %) |
|  | >15 | 10 (4 %) |
| **Status at discharge** | Alive | 237 ( 89%) |
|  | Dead | 30 (11%) |

9. Standardize the description/name of your n=185 Severe Illness group. Table 1 lists Co-morbidities at admission (n=23) and Figure 1 describes Common Complications during period of admission (n=185). Later in Comparison of Direct Costs Incurred by Patient Characteristics it states patients with co-morbidities incurred higher costs than those with no comorbidities and you are talking about your n=185 group but it could be misconstrued as your n=23 group. The Common Complications figures are never referred to as comorbidities but the n=23 group is referred to as comorbidities.

Response: Comorbidities were long standing illnesses that the patient already had at the time of admission. These patients with comorbidities were 23. Complications during admission were illnesses associated with measles and these were 185.

In comparison of direct costs incurred by patient characteristics, we used those with co-morbidities, and not those with complications.

10. Regarding Common Complications, you have information of patients who died (n=30), but it would also be helpful regarding Common Complications (n=185) to have information of immunization status.

RESPONSE: For those who had multiple complications (n=185), their immunization status was as follows, and this has been shared as supplementary information, S2

| **Immunization status** | **Frequency** | **Percentage** |
| --- | --- | --- |
| Yes | 50 | 27.0% |
| Unknown | 48 | 25.9% |
| Not due | 33 | 17.8% |
| No | 54 | 29.2% |
| **Grand Total** | **185** | **100.00%** |

11. For Duration of Hospital Stay and Characteristics Among the 60 Patients Who Stayed Longer Than 7 days, you say 207 (77%) stayed <7 days and 60 (13%) stayed >7 days, however 77%+13% does not equal 100%. Later in Table 3 you present information on patient/s who stay >15 days but this group is not mentioned in the Characteristics text section. Please give the n of patients who stay >15 days.

Response: This has been clarified in table 1 in the results section. 207 patients stayed 0-7 days, 50 patients stayed 8-15 days, and 10 patients stayed more than 15 days.

Patients who stayed over 15 days in the hospital incurred a higher average cost; is that just a cumulative effect of the number of days, or is their daily rate higher?

Response: Patients who stayed over 15 days incurred a higher average cost and this cost was a cumulative effect of the number of days.

And of the participants who stay >7 days, a majority were less than one year; this brings up topics around the age of immunization initiation which is never brought up in the discussion.

Response: We agree that among those who stayed > 7days, majority were less than 1 year old. We however feel that going into the detail of immunization scheduling would mean diversion from the study objective.

12. Table 2 lists “Treatment” costs. What are Treatment costs? Drugs? The Study Procedure section says direct health care costs are drugs, supplies, and investigations, but Table 2 lists direct health care costs as Blood Test, Radiology, and Treatment. Please clarify terminology and how costs were estimated and categorized.

Response : This has been clarified in paragraph 3 of the study procedure section. Treatment costs include cost of drugs and supplies. Direct health care costs include treatment costs (drugs and supplies) and investigations (blood tests and radiology tests)

13. Table 3 displays that immunized and non immunized patients cost the hospital the same amount. Supplemental Table 1 does establish that 13/30 of the high-cost-Dead at Discharge individuals are non immunized, but no analysis is done of the 185 high-cost-Comorbidity individuals regarding immunization status.

Response: For those who had multiple complications (n=185), their immunization status was as follows. This table is shared as supplementary information in the new submission.

| **Immunization status** | **Frequency** | **Percentage** |
| --- | --- | --- |
| Yes | 50 | 27.0% |
| Unknown | 48 | 25.9% |
| Not due | 33 | 17.8% |
| No | 54 | 29.2% |
| **Grand Total** | **185** | **100.00%** |

14. Many comparisons with Pakistan but no context to similarity of hospitals, health systems, etc.

Response: The issue on similarity of hospitals has now been addressed in the discussion. These hospitals are similar to Mulago as they are referral and teaching facilities, offering quite similar services. We however agree that there are likely to be important contextual and health system differences in the two settings. Unfortunately, we have not come across similar studies to our own in facilities more comparable to Mulago hospital.

15. Do not refer to participants as ‘babies’, inclusion criteria was 0-12 years.

Response: We appreciate the reviewer for this observation. The word “babies” has accordingly been replaced with “participants”.

16. It is stated that immunization costs are less than a dollar and hospital treatment costs are high, “this is strong ground to base on to promote vaccination,” however the data in this study showed no difference in hospital spending when comparing immunized vs. non immunized.

Response: We did not necessarily expect hospital costs to be different comparing the immunised and unimmunised participants. We however expect that immunisation should reduce the incidence of measles infection (clinical measles), and thereby reduce health expenditure due to measles.

17. “Since vaccines are available at health facilities, it is clear that the most important challenge is related to utilization/demand.” There is no basis for this statement, no regard or discussion of societal and economic barriers to health facility access.

Response: We agree with the reviewer and have deleted this statement from the discussion.

- Conclusion:
  18. “Efforts are needed to promote immunization against measles and related mortality and thus reduce costs to the healthcare delivery system.” The data presented in this paper does not support this conclusion. A more accurate conclusion states that complications/comorbidities are present in a majority of fatal measles cases and comorbid cases carry a higher cost to the healthcare system.

Response: We agree with the reviewer, and the conclusion has been edited to read: “Complications are common in majority of fatal measles cases and these carry a high cost to the healthcare system”.

Reviewer #3: This manuscript describes the burden of measles in hospitalized cases in a Ugandan national hospital. The costs of medical care and hospitalization are calculated and described. The authors analyze the factors associated with increased hospital cost. Overall, this paper is structured well. The English writing is adequate. The data combine clinical information and cost, thus providing useful information for their country’s national program to improve vaccination coverage and follow up.

There are both UK and American English spellings in the paper that need to be resolved. Be careful to make the discussion and conclusions based on the data and not overly speculative.

Response: The discussion and conclusion have been edited based on the data presented.

Abstract
No comments here, but some changes may be needed that are related to comments in the body of the manuscript.

Introduction
Overall: Suggest to include a map to show the percentages of measles vaccination and where the outbreaks are.

Response: Whereas this would have been very useful information to present, we are unfortunately unable to find a map showing the distribution of previous outbreaks and coverage of vaccination in the country.

“Besides morbidity and mortality, costs related to treating a child with measles pose a big burden to the family and the already financially stretched health care delivery system, and are increased when the measles is complicated.” It would be helpful to give some details of what defines measles complications.

Response: Measles related complications include, pneumonia, encephalitis, malnutrition, diarrhea, blindness, croup, otitis media among others; further details are provided in paragraph 4 of the introduction.

Methods
Study design and setting: please describe the national guidelines for measles vaccine in your setting.

Response: In Uganda, Measles vaccine is given at 9 months, and is freely available at all government health facilities as well as some private facilities. In times of epidemics, the vaccine is also often administered widely through mass vaccination campaigns. This has been added to the study design and setting section.

“Those with incomplete data like undocumented inpatient number, age,and sex were excluded.” List all inclusion and exclusion critiera.

Response: The full eligibility criteria has been added in the second paragraph of the study procedures section. Inclusion criteria was all available retrieved charts of children 0-12 years admitted with clinical measles from 1^st^January 2018 to 31st December 2018. Exclusion criteria included a chart with a documented diagnosis of measles and yet does not have the clinical definition of measles as fever, maculopapular rash with either cough, coryza or conjunctivitis was excluded. All charts without documented, inpatient number, age, sex, were excluded. Those charts with illegible handwriting were also excluded.

A trial diagram should be presented and I suggest that the authors use the STROBE checklist to confirm their work.

Response: We appreciate the reviewer for this suggestion. A flow chart has now been added to the results section of the revised manuscript as Figure 1.

“The first chart was selected using simple random sampling.” Give the details of the random sampling rather than using the word ‘simple’.

Response: The sampling technique used was systematic sampling and the details have been provided as follows “Using systematic sampling technique, the sampling frame was 536 charts and the sample size of 267 was calculated using the formula for fixed populations. We obtained a skip interval (k=2), and every second chart was chosen until the required sample size was achieved”. The first chart was chosen randomly from the charts as a starting point.

These details have been provided under the study procedure section.

“Information abstracted from the patient’s charts included: Demographics (age, gender)….” If you mean male or female, then ‘sex’ will be more precise than ‘gender’, as gender has many categories.

Response: The word gender has been edited to “sex” in the study procedure section.

“These were checked against the patient’s record daily for completeness and accuracy.” I do not understand why the DAF's were checked against the patient record every day. Is this not a retrospective study?

Response: The word ‘daily’ had been included erroneously, and has been deleted.

“School of Medicine Research and Ethics Committee (SOMREC) and The Mulago Hospital Institution Review Board.” Provide the ethical approval number of the EC.

Response : The ethical approval number of the committee is REF 2019-009, and this has been added to the ethics approval and consent section.

Results
“A total of 570 patients were admitted between 1st January and 31st December 2018
with a clinical diagnosis of Measles and we managed to retrieve 553…” Measles should not be capitalized. '...we managed to retrieve....' could be changed to something more objective such as ' ..... and 553 charts were retrieved...

Response: This has been edited in the first paragraph of the results section to read: A total of 570 patients were admitted between 1^st^ January and 31^st^ December 2018 with a clinical diagnosis of measles and a total of 553 (97%) charts were retrieved.

Table 1: Definitions of Immunization status need to be more clear. Does 'yes' mean up to date or had at least one vaccine? Does 'no' mean never before or due? Does 'Not due' mean up to date
I also suggest to present whole percentages as the addition of the decimal point does not add any more precision.

Response: In table 1, immunization status ‘Yes’ means-received the measles vaccine, ‘No’ means-did not receive the measles vaccines, ‘Not due’ means less than 9months, “Unknown” means not recorded. Further details are found in the Data abstraction form which has been submitted as supplementary information (S1).

Do immunizations include any vaccines or only measles vaccine?

Immunisations means only measles vaccine in this study.

It would be interesting to know how many were too young to have received measles vaccine, how many were eligible but did not receive measles vaccine.

Response: Fifty-six patients were too young to have received measles vaccine. Seventy-nine were eligible but did not receive the measles vaccine. These details have been provided in table 1 under the immunization status

This is important because the infants who were not eligible for measles vaccine (due to their age) should not be included in the cost analysis as measles vaccine access is not the issue. This must be specified in the study methods.

Response: We think it is still useful to show the economic burden of managing a child with measles, including those that are below the cut off age for administration of the measles vaccine in our setting. There is still debate on the possibility of considering vaccinating babies for measles earlier than 9 months, especially in a setting like ours where epidemics are common. It would be good to carry out a cost benefit analysis of this intervention, and we think that our findings may be useful in that regard.

I also suggest to present whole percentages as the addition of the decimal point does not add any more precision.

Response whole percentages have been presented in table 1.

| **Immunization status** | Yes | 63 (24%) |
| --- | --- | --- |
|  | No | 79 (30%) |
|  | Unknown | 69 (26%) |
|  | Not due | 56 (21%) |
| **Co-morbidities at admission** | Yes | 23 (9%) |
|  | No | 244 (91%) |

“Out of these, 17 (57%) were male. 21 (70%) of these were aged between 0-1 years and 13 (44%) were not immunized.” Of the ones not immunized, how many were <12 months (or 9 months) and not yet eligible for vaccination?

Response; ‘not immunized’ means never received the measles vaccine though was due. Six participants (20%) were less than 9 months and therefore not yet due for measles vaccination. Further details are provided in table 1

Figure 2: Please clean up the x-axis labels. There should be no underscores and PTB needs to be defined.

Response: Thanks for these suggestions. This has been done.

“The median length of hospital stay among children admitted with measles was 4.0 (IQR
3.0-7.0) days.” This result is already mentioned above. Please report it only once.

Response: This repletion has been deleted.

“Of these, 46 (77 %), were less than one year and 36….” Of which one - the ones that stayed less than or more than 7 days?
Response; Of the 60 who stayed longer than 7 days,46 (77%) were less than one year and 36 were male.

Table 2: Clean the Item names (ie, Maintenance)

“Male patients incurred higher average costs (36.08USD, SD=35.09) than their female
counterparts (30.24USD, SD=24.89), (F=6.126, P=0.011).” There may be correlating variables here. For example, were the patients with co-morbidities more likely to have complications? Were males more likely to have co-morbidities? Hospital duration should be included here. Please perform either a multivariable ANOVA or linear regression that includes all of the potentially related variables in the model. This also would need to be described in the statistical section.

Response: MANOVA and COVARIANCE test was conducted and the findings revealed no significant interaction between independent variables (See appendix table at the end of this document; not included in the manuscript)

“Patients who stayed for over 15 days in the hospital incurred higher average costs….than those who stayed between 8-15 days….” The reader does not know how many persons stayed >15 days. This should be presented above. I would suggest using similar groupings and the same definition as what were presented above (more than 7 days).

Response: The categorization has been revised as suggested by the reviewer, and frequencies added.

Table 3: Double check all the numbers in this table. All the standard deviation values seem too high and definitely should not greater than the mean. The number of deaths does not match the text, where around 10% of the cases had died (not 45% as is stated in this table). Hospital stay and complications may be collinear - this probably should be addressed in the results. Once you have shown that they are collinear, perhaps only one of them (either hospital stay or complications) needs to be presented.

RESPONSE: All the figures have been revisited and revised accordingly (See table 3)

Discussion
“Mortality was found highest among the children who were not immunised, possibly
because they developed severe complications.” With your data, you can run this analysis using a multivariable ANOVA or logistic regression model (death yes or no) which includes the related variables (sex, co-morbidities, hospital stay/complications, immunization status, etc).

Response: It was indeed revealed that there was a significant association between immunization status and status at discharge and by comparison of proportions for those who died, a large proportion of those who were not immunized (43.3%) died.

|  | **Status of discharge** | |  |  |
| --- | --- | --- | --- | --- |
| **Immunization status** | **Alive** | **Died** | **Pearson chi-2** | **P-value** |
| No | 66 (27.8%) | 13 (43.3%) | 6.176 | 0.037 |
| Not due | 50 (21.1%) | 6 (20.0%) |  |  |
| Unknown | 62 (26.2%) | 7 (23.3%) |  |  |
| Yes | 59 (24.9%) | 4 (13.3%) |  |  |

“…..was on average 122,500
Ugandan shillings which is approximately 33.13 US dollars (1 US Dollar=3697.92 UGX) [17], much of which goes to treatment and supplies.” How much goes to treatment and supplies? (A percentage would be interesting here).
Response: This has been edited to include the percentage. 66.3% of the 33.13 US dollars goes to treatment.

“Such costs are far beyond the cost required to immunise a child against measles which the World Health Organisation estimates to be less than a dollar.” How much is a measles vaccine in your setting?
Response: Measles vaccine in Uganda is provided free of charge in public facilities. This information has been added to the study design and setting section.

“The possible explanation for this is that those with co-morbidities had to be managed for both measles in addition to other co-morbidities.” Is it more expensive because they had higher risks for complications and stayed in the hospital longer? This will need to be addressed in the analysis and results.

“Patients who also stayed longer in hospital (> 15 days) incurred higher average costs than those who stayed for less days.” Please refer to the comment previous. Complications, co-morbidities and hospital stay are likely related. This needs to be addressed in the analysis.

Response: From the table in of MANOVA AND COVARIANCE (at the end of this document), there was no significant interaction between length of hospital stay and co-morbidities.

“There is therefore a possibility that some diagnoses of measles were
actually other viral exanthems like rubella, scarlet fever, varicella and roseola.” Suggest specifically stating that measles serology was not performed.

Response: Measles serology was not done. There is therefore a possibility that some diagnoses of measles were actually other viral exanthems like rubella, scarlet fever, varicella and roseola. This has been edited in the discussion under limitations.

“We instead assumed that they were received since they would ideally be required
for management of these cases.” This is an interesting point especially to readers who live in cultures where this occurs less often or rarely. It might be worthwhile adding it to your statistical section or study methods – specifically stating that you are assuming all costs incurred were received by the patient.

Response: This has been added under the study procedure. All costs incurred were assumed to have been received by the patient since they would be required in the management of measles.

“The estimated average direct health care cost of treating a measles case in the National Referral Hospital was considerably high.” I suggest adding a 'cost savings' value. For example, of the cases in this publication, if 90% (since a single dose of MMR vaccine should result in 90% of the population to be immune) hospitalizations (xx numbers of cases) were prevented with measles vaccination, then xxx dollars (total cost minus the vaccine cost) would have been saved by the health system.
Response: While we agree that the extra analyses suggested by the reviewer are useful, our study does not have all the information to be able to calculate the costs that would be averted by vaccination. This would have been possible if we had also determined other costs, such as those incurred by the family in managing a child with measles. We were unable to determine these costs because of the retrospective nature of our study,

Acknowledgements
“…and the participants whose charts we used.” Suggest changing the word ‘charts’ to ‘information’

This has been edited to “we acknowledge ---and the participants whose information was used.”

**APPENDIX:** **MANOVA AND COVARIANCE**

| *Tests of Between-Subjects Effects* | | | | | |
| --- | --- | --- | --- | --- | --- |
| Dependent Variable: TOTAL_COST | | | | | |
| Source | Type III Sum of Squares | df | Mean Square | F | Sig. |
| Corrected Model | 2426947532410.275^a^ | 38 | 63867040326.586 | 15.412 | .000 |
| Intercept | 53283256741.427 | 1 | 53283256741.427 | 12.858 | .000 |
| Hosp_days | 1719512911076.266 | 1 | 1719512911076.266 | 414.935 | .000 |
| Status_disch | 14106345458.013 | 1 | 14106345458.013 | 9.404 | .006 |
| Child_sex | 11208229002.581 | 1 | 11208229002.581 | 12.705 | .001 |
| Immun_status | 19838439780.622 | 3 | 6612813260.207 | 1.596 | .191 |
| Comorbid | 11159638280.231 | 1 | 11159638280.231 | 11.693 | .002 |
| Age_grp | 1882180887.279 | 1 | 1882180887.279 | .454 | .501 |
| Status_disch * Child_sex | 32028780916.747 | 1 | 32028780916.747 | 3.729 | .063 |
| Status_disch * Immun_status | 28075718477.306 | 3 | 9358572825.769 | 2.258 | .082 |
| Status_disch * Comorbid | 42949737442.758 | 1 | 42949737442.758 | 2.364 | .071 |
| Status_disch * Age_grp | 468108717.573 | 1 | 468108717.573 | 0.113 | .737 |
| Child_sex * Immun_status | 14576723137.835 | 3 | 4858907712.612 | 1.173 | .321 |
| Child_sex * Comorbid | 916677575.617 | 1 | 916677575.617 | .221 | .639 |
| Child_sex * Age_grp | 312891539.931 | 1 | 312891539.931 | .076 | .784 |
| Immun_status * Comorbid | 26248021092.341 | 3 | 8749340364.114 | 2.111 | .100 |
| Immun_status * Age_grp | 1203534933.753 | 2 | 601767466.877 | .145 | .865 |
| Comorbid * Age_grp | 2067034661.382 | 1 | 2067034661.382 | .499 | .481 |
| Status_disch * Child_sex * Immun_status | 7933046397.728 | 2 | 3966523198.864 | .957 | .386 |
| Status_disch * Child_sex * Comorbid | 8190966419.017 | 1 | 8190966419.017 | 1.977 | .161 |
| Status_disch * Child_sex * Age_grp | 1654528454.945 | 1 | 1654528454.945 | .399 | .528 |
| Status_disch * Immun_status * Comorbid | 5832332433.941 | 1 | 5832332433.941 | 1.407 | .237 |
| Status_disch * Immun_status * Age_grp | 5288311176.002 | 1 | 5288311176.002 | 1.276 | .260 |
| Status_disch * Comorbid * Age_grp | .000 | 0 | . | . | . |
| Child_sex * Immun_status * Comorbid | 23304054868.832 | 1 | 23304054868.832 | 2.623 | .109 |
| Child_sex * Immun_status * Age_grp | 13511495227.277 | 2 | 6755747613.638 | 1.630 | .198 |
| Child_sex * Comorbid * Age_grp | .000 | 0 | . | . | . |
| Immun_status * Comorbid * Age_grp | .000 | 0 | . | . | . |
| Status_disch * Child_sex * Immun_status * Comorbid | .000 | 0 | . | . | . |
| Status_disch * Child_sex * Immun_status * Age_grp | .000 | 0 | . | . | . |
| Status_disch * Child_sex * Comorbid * Age_grp | .000 | 0 | . | . | . |
| Status_disch * Immun_status * Comorbid * Age_grp | .000 | 0 | . | . | . |
| Child_sex * Immun_status * Comorbid * Age_grp | .000 | 0 | . | . | . |
| Status_disch * Child_sex * Immun_status * Comorbid * Age_grp | .000 | 0 | . | . | . |
| Error | 944845015622.405 | 228 | 4144057086.063 |  |  |
| Total | 7378510053181.000 | 267 |  |  |  |
| Corrected Total | 3371792548032.680 | 266 |  |  |  |
| a. R Squared = .720 (Adjusted R Squared = .673) | | | | | |
