# Supplementary material for: The immediate treatment outcomes and cost estimate for managing clinical measles in children admitted at Mulago Hospital: A retrospective cohort study
Source: PLOS Glob Public Health. 2023 Jul 21;3(7):e0001523. doi: 10.1371/journal.pgph.0001523 (PMC10361502; doi:10.1371/journal.pgph.0001523)
Supplement: Attachment — Submitted filename: Response to reviewers.docx [file pgph.0001523.s006.docx]

**15/06/23**

**The Editor,**

**PLOS Global Public Health Journal**

RE: Resubmission of an original article titled **“The immediate treatment outcomes and cost estimate for managing clinical measles in children admitted at Mulago Hospital: a retrospective cohort study”**

We are delighted that PLOS Global Public Health considered our manuscript for review. We have given careful consideration to the comments from the reviewers and editor, and have addressed them all.

Attached is a point-by-point explanation of how we have addressed the concerns and revised our manuscript.

We look forward to a favorable opinion from the editor.

Yours sincerely,

Dr Namugga Barbara,

on behalf of all the authors

Response to reviewers

Journal Requirements:

1. Please review your reference list to ensure that it is complete and correct. If you have cited papers that have been retracted, please include the rationale for doing so in the manuscript text, or remove these references and replace them with relevant current references. Any changes to the reference list should be mentioned in the rebuttal letter that accompanies your revised manuscript. If you need to cite a retracted article, indicate the article’s retracted status in the References list and also include a citation and full reference for the retraction notice.

Response: We have reviewed the reference list and it is complete. We have also not cited any response that has been retracted.

2. Please amend your detailed Financial Disclosure statement. This is published with the article. It must therefore be completed in full sentences and contain the exact wording you wish to be published.

a. State the initials, alongside each funding source, of each author to receive each grant.

University of Minnesota funded this study with 270 US dollars ( 1million Uganda shillings) towards data collection.
b. State what role the funders took in the study. If the funders had no role in your study, please state: “

The funders had no role in study design, data collection and analysis, decision to publish, or preparation of the manuscript.”
c. If any authors received a salary from any of your funders, please state which authors and which funders.

The authors did not receive any salary from the funders

If you did not receive any funding for this study, please simply state: “The authors received no specific funding for this work.”

Additional Editor Comments (if provided):

We appreciate your edits to the manuscript. I have just a few minor issues to address.

Could you provide more detail about the sample size? You mention sample frame and that you only needed half of that, but could you explain why? (for instance, something like: "to estimate [either a relationship or a statistic] we would need a sample size of XXXX with an alpha of 0.05 and a power of 80%

Response; Sample size was determined based on the formula by Yamane, (1967);

$n_{r}=\frac{N}{{1+N(e)}^{2}}$

where N is the sampling frame estimated at 812 from the Mulago Hospital medical admission records (HMIS inpatient register) and e is the allowable error, taken as 5%. The sample size used 267 patient records.

systemic sampling technique was used to pick the charts.using a skip interval k=2,every 2^nd^ chart was chosen till the required sample size of 267.

Could you edit the figures? In essence, try to make them have higher quality in terms of DOI and be conscientious of how you use screenshots. For one, remove the spell check so there aren't wiggly lines underneath the text. I believe you are using Microsoft Office. Some people have found ways of exporting higher quality images (for instance, see <https://learn.microsoft.com/en-us/office/troubleshoot/powerpoint/change-export-slide-resolution>) using PowerPoint. If you have another method, that possibly could work.

Response: The figures have been edited and the quality has been improved.
